# A novel model predicted liver cirrhosis constructed by ultrasound and serological in autoimmune liver hepatitis

**DOI:** 10.1097/MD.0000000000035295

**Published:** 2023-09-22

**Authors:** Siyi Feng, Haibin Tu, Lihong Chen

**Affiliations:** a Department of Ultrasound, Mengchao Hepatobiliary Hospital of Fujian Medical University, Fuzhou, Fujian, China.

**Keywords:** autoimmune liver hepatitis, cirrhosis, noninvasive model, prediction, serology, ultrasound

## Abstract

To establish a noninvasive model based on two-dimensional shear wave elasticity (2D-SWE) technology, ultrasound feature and serological indicators to predict cirrhosis in autoimmune hepatitis (AIH) and verified. Patients with AIH confirmed by liver biopsy with liver ultrasound and serological examination were collected from January 2019 to May 2022. Patients were divided into cirrhosis and non-cirrhosis groups. Basic indexes, ultrasound indexes and serological indexes were collected. Multivariable logistic regression used for screening independent risk factors predicting cirrhosis, construct the AIH cirrhosis prediction model, named autoimmune hepatitis cirrhosis (AIHC). Determine best cutoff score according to the Youden index, verified the model’s predictive efficacy. One hundred forty-six patients were collected. The following indicators were independent risk factors for predicting cirrhosis: LS (OR: 1.416, *P* = .015), splenomegaly (OR: 10.446, *P* = .006), complement C4 (OR: 0.020, *P* = .009). The best cutoff score was 65, with a sensitivity 88.9% and specificity 75.6%; the area under curve was 0.901, AIHC possessed a higher net reclassification index (NRI) and integrated discrimination improvement compared with other indexes, and AIHC had the best clinical decision curve. The AIHC constructed in this study has better predictive efficacy than other noninvasive indexes, and we visualized the model for easy application, which was worth further promotion in clinical practice.

## 1. Introduction

Autoimmune hepatitis (AIH) is a liver-related disease characterized by autoimmune response in genetically predisposed individuals. The global morbidity is about 1.3 to 9.4/100,000.^[1]^ The clinical characterized by elevated serum aminotransferase and immunoglobulin G levels, seropositive results for autoantibodies and moderate to severe interface hepatitis in histologic findings. Histological confirmation of liver biopsy samples is required for diagnosis, as is exclusion of hepatitis associated with viral infections, primary biliary cholangitis, primary sclerosing cholangitis, drug-induced liver injury and Wilson disease. The International Autoimmune Hepatitis Group has issued guidelines for the diagnosis of AIH in adults, whereas the European Society of Paediatric Gastroenterology, Hepatology and Nutrition has proposed a scoring system for AIH in children.^[2]^ AIH usually requires lifelong treatment, but in the stage of cirrhosis, when the liver response to various drugs is significantly reduced. Therefore, it is of great significance to know whether the patient is in the stage of cirrhosis and prompt adjustment of the treatment plan is required.

Although liver biopsy is regarded as the gold standard of liver fibrosis grading, there are also shortcomings with it, such as high cost and limited materials, which cannot represent the condition of the whole liver, and may lead to serious complications after biopsy. With the popularization of noninvasive concepts, more noninvasive indicators are used to assess the grade of liver fibrosis.^[3–6]^ Aspartate aminotransferase-to-platelet ratio index (APRI) and FIB4 ([age × AST] ÷ [PLT × ALT]) are the most commonly used serological indexes.^[7,8]^ Ultrasound examination indexes include elasticity,^[9]^ liver capsule smoothness, gallbladder wall smoothness, splenomegaly and so on.^[10,11]^ There is a lack of stability with serological indexes, and the ultrasound indexes can reflect the general changes of the liver, but they do not evaluate the function of the liver. Therefore, it is a creative idea to combine the two to build a prediction model.

## 2. Materials and Methods

This study was reviewed and approved by the Mengchao hepatobiliary hospital ethics committee (ethical number 2021-088-01).

### 2.1. Patients

In this study, 1526 patients who underwent liver biopsy, ultrasound examination and serological examination in our hospital from January 2019 to May 2022 were initially included, and 146 patients were finally included after screening according to the following criteria. General information about the patient was collected: height, weight, body mass index (BMI), gender, age, history of diabetes.

Inclusion criteria: (1) AIH diagnosed by liver biopsy for the first time; the inflammation grading is lower than level 2; (2) no other treatment was performed before biopsy, including antifibrotic treatment and traditional Chinese medicine treatment; (3) complete data; (4) obtain the consent of the patient or guardian. (5) Patients age was 18 to 80 years.

Exclusion criteria: (1) pathological results showed viral infection (hepatitis B, hepatitis C, etc); (2) treatment history of AIH; (3) incomplete pathological data (lack of liver ultrasound examination indexes or serological indexes).

### 2.2. Ultrasound parameters

**Figure 1.**
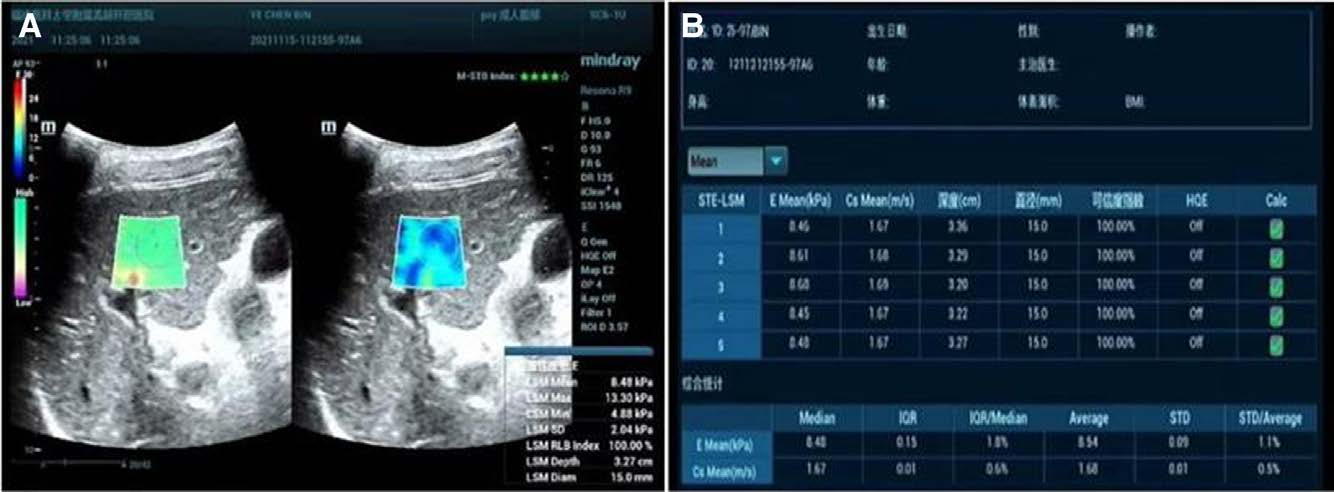
Measurement of liver stiffness in patients. (A) A 67 years old female with AIH, two-dimensional images combined with liver stiffness measurement; (B) analysis of measurement results, with the median (11.19 Kpa) as the final collected data.

The Mindray Resona R9 ultrasonic diagnostic apparatus was used, with an abdominal convex array probe and a probe frequency of 2 to 6 MHz. Before the examination, the patient underwent fasting for 8 hours. The patient was instructed to lie flat and lift both upper limbs to fully expose the skin of the upper abdomen. Firstly, the routine abdominal ultrasound examination was performed. The following indicators were collected: the oblique posterior diameter of the right liver (>14 cm) was judged as hepatomegaly. If it is <10 cm, it is judged as liver shrinkage. Liver capsule (subjective evaluation of smoothness), liver parenchyma echo (subjective evaluation of uniformity), portal vein diameter (measured at about 1 cm from the hepatic portal), gallbladder wall (subjective judgment of smoothness), spleen size (when the long diameter is >12 cm and the thick diameter is >4 cm, it is judged as splenomegaly). Collateral circulation (left gastric vein, umbilical vein, splenorenal shunt vein, when any blood vessel is detected, collateral circulation is judged to be formed). Liver stiffness (LS) measurement was performed in two-dimensional shear wave elasticity mode, avoiding intrahepatic vessels as much as possible. The sampling frame was 3 cm × 3 cm in size and placed at the pre-biopsy site, so that the measured hardness value could reflect the real situation of the biopsy tissue to the greatest extent. Before measurement, ask the patient to hold his breath. After the image gets stable, activate the sampling procedure. When the sampling frame is completely filled with color, set the measurement diameter to 1.5 cm, measure 5 times continuously at different parts of the sampling frame, control the percentile difference within 30% IQR, and take the median as the final result. The measurement diagram is shown in Figure 1. The above operations are undertaken by doctors with more than 10 years of experience in ultrasound.

### 2.3. Serological indicators

After fasting for 8 hours, blood in the upper limb venous was collected, and the following indicators were collected: alanine aminotransferase (ALT), aspartate aminotransferase (AST), platelet (PLT), glutamyl transpeptidase (GGT), albumin (ALB), cholesterol, total bilirubin, prothrombin time, prothrombin time activity, alkaline phosphatase, international normalized ratio (INR), complement C3, complement C4, Golgi protein-73.

### 2.4. Noninvasive indicators

According to previous literature reports, the following noninvasive indicators were included in this study as comparative objects.^[12]^

(1) APRI: AST/upper limit of normal/PLT × 100; (2) BARD: BMI ≥ 281, AST/ALT ≥ 0.82, history of diabetes mellitus 1; (3) FIB-4: (age × AST) ÷ (PLT × ALT); (4) NF: –1.675 + 0.037 × age + 0.094 × BMI + 1.13 × diabetes history (yes = 1, no = 0) + 0.99 × AST/ALT-0.012 × PLT-0.66 × ALB; (5) Forns index: 7.811 to –3.131 × lnPLT + 0.781 × lnGGT + 3.467 × ln age-0.014 × cholesterol; (6) GPR: GGT/PLT; (7) Lok Index: –5.56 to −0.0089 × PLT + 1.26 × AST/ALT + 5.27 × INR; (8) AAR: AST/ALT.

### 2.5. Pathological diagnostic criteria

In this study, Metavir grading method was used to stage liver fibrosis. Fibrosis grade (S) was divided into S0–S4. Cirrhosis was diagnosed when the fibrosis grade S = 4, and the remaining cases were diagnosed as non-cirrhosis.

### 2.6. Statistical methods

The measurement data were presented in the form of mean ± standard deviation, and the enumeration data were presented in the form of percentage. If the measurement data met the conditions of normality and homogeneity of variance, the 2 independent samples *t*-test were used, otherwise, the 2 independent samples Mann–Whitney rank sum test was used. Independent risk factors for the AIH cirrhosis were selected by multivariate logistic regression analysis, and a nomogram model (autoimmune hepatitis cirrhosis, AIHC) of the AIH cirrhosis was constructed. The internal validated in the model by Bootstrap method, and the receiver operating characteristic curve, drawing the area under the curve of each index was compared by Delong method to look for differences. The optimal cutoff value was obtained according to Youden index. Calibration curve and clinical decision curve were drew to evaluate calibration degree and the clinical net benefit of the model. Two-sided test was used for the above tests, and *P* < .05 was considered as statistically significant. The statistical software used in this study is R (4.0.1).

## 3. Results

### 3.1. General information of the patients

General information of all enrolled patients (Table 1). Among 146 patients, there were 27 patients in the cirrhosis group and 119 patients in the non-cirrhosis group, with a mean age of 52.4 ± 10.1 years. The proportion of splenomegaly and liver stiffness (LS) in the cirrhosis group were higher than those in the non-cirrhosis group. LS, hepatic echo, splenomegaly, GP73, total bilirubin, prothrombin time, prothrombin time activity and INR in the cirrhosis group were higher than those in the non-cirrhosis group, while ALB, PLT count and complement C4 in the cirrhosis group were lower than those in the non-cirrhosis group. There were significant differences in the above indexes between the 2 groups.

**Table 1:** Basic characteristics.

Index	Total (n = 146)	Non-cirrhosis (n = 119)	Cirrhosis (n = 27)	*P*-value
Age (years mean, SD)	52.4 ± 10.1	52.2 ± 10.5	53.2 ± 8.0	.65
Gender				
Female	129 (88.4%)	105 (88.2%)	24 (88.9%)	1
Male	17 (11.6%)	14 (11.8%)	3 (11.1%)	
BMI	22.4 ± 2.6	22.6 ± 2.5	21.7 ± 2.8	.021
Right-lobe-size				
Normal	135 (92.5%)	113 (95.0%)	22 (81.5%)	.031
Shrink	11 (7.5%)	6 (5.0%)	5 (18.5%)	
Liver echo				
Normal	77 (52.7%)	68 (57.1%)	9 (33.3%)	.018
Coarse	49 (33.6%)	39 (32.8%)	10 (37.0%)	
Node	20 (13.7%)	12 (10.1%)	8 (29.6%)	
Capsual				
Smooth	128 (87.7%)	106 (89.1%)	22 (81.5%)	.33
Rough	18 (12.3%)	13 (10.9%)	5 (18.5%)	
Collateral				
Normal	146 (100.0%)	119 (100.0%)	27 (100.0%)	1
Collateral	0 (0.0%)	0 (0.0%)	0 (0.0%)	
Liver stifness(kpa)	7.4 ± 4.2	6.4 ± 3.1	12.0 ± 5.4	<.001
Portal venous(cm)	1.1 ± 0.1	1.1 ± 0.2	1.1 ± 0.1	.003
Spleen				
Normal	101 (69.2%)	92 (77.3%)	9 (33.3%)	<.001
Splenomegaly	45 (30.8%)	27 (22.7%)	18 (66.7%)	
Gallbladder				
Smooth	51 (34.9%)	45 (37.8%)	6 (22.2%)	.18
Rough	95 (65.1%)	74 (62.2%)	21 (77.8%)	
ALT(U/L)	135.7 ± 545.9	145.7 ± 601.4	91.9 ± 134.7	.76
AST(U/L)	80.5 ± 64.7	76.9 ± 60.3	96.5 ± 80.7	.079
PLT(/L)	231.3 ± 67.8	239.3 ± 64.5	195.8 ± 71.8	.001
ALB(g/L)	40.3 ± 20.6	41.7 ± 22.4	34.3 ± 6.3	<.001
GGT(u/L)	304.3 ± 306.3	313.2 ± 325.6	265.2 ± 200.1	.73
CHO(mmol/L)	3.42 ± 1.14	3.46 ± 1.09	3.38 ± 1.02	.82
TBIL(umol/L)	36.4 ± 55.2	32.1 ± 53.5	55.3 ± 59.3	.018
ALP(mmol/L)	284.4 ± 228.3	275.8 ± 232.7	322.3 ± 207.2	.16
GP73(ug/L)	102.4 ± 51.0	89.4 ± 20.7	159.8 ± 91.5	<.001
PT(s)	12.3 ± 1.1	12.1 ± 0.9	13.2 ± 1.7	<.001
PTA(%)	108.2 ± 19.9	109.7 ± 19.9	101.6 ± 19.1	.016
INR	1.0 ± 0.2	1.0 ± 0.1	1.1 ± 0.4	.011
C3(g/L)	1.2 ± 0.3	1.2 ± 0.3	1.2 ± 0.3	.71
C4(g/L)	0.2 ± 0.1	0.2 ± 0.1	0.1 ± 0.1	<.001

ALB = albumin; ALP = alkaline phosphatase; ALT = alanine aminotransferase; AST = aspartate aminotransferase; BMI = body mass index; C3 = complement C3; C4 = complement C4; CHO = cholesterol; GGT = glutamyl transpeptidase; GP-73 = Golgi protein-73; INR = international normalized ratio; PLT = platelet; PT = prothrombin time; PTA = prothrombin activity; TBIL = total bilirubin.

### 3.2. Relevance of each indicator

**Figure 2.**
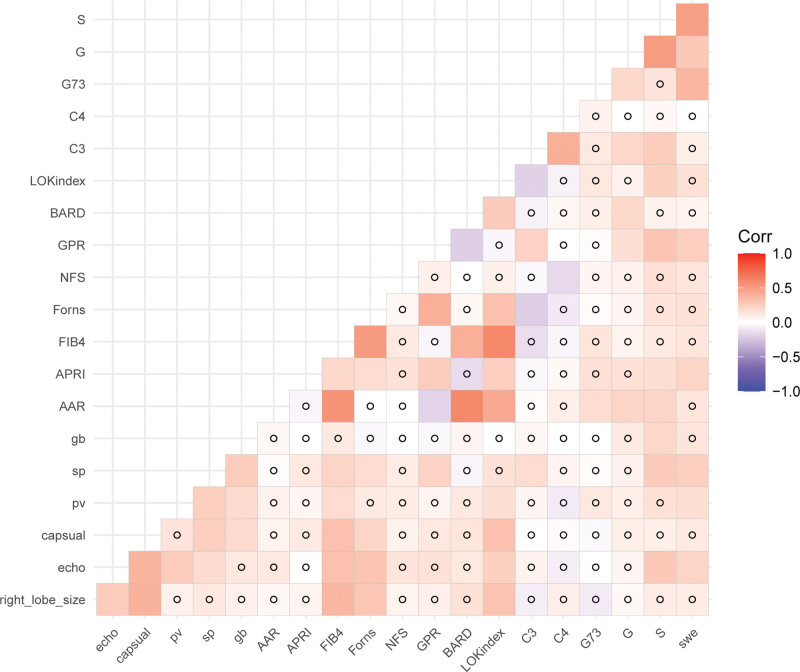
Correlation heat map of each index. S: liver fibrosis stage, G: inflammation stage, swe: liver stiffness, laminin: laminin, PIIIP: type III procollagen telopeptide, HA: hyaluronidase, echo: liver echo, right-lobe-size: right hepatomegaly, sp: splenomegaly, PV: portal vein diameter, collateral: collateral formation. Circle means no correlation.

Correlation of each index (Fig. 2). The indexes significantly correlated with the stage of liver fibrosis were LS (*R* = 0.493, *P* < .001), Lokindex (*R* = 0.244, *P* = .003), GPR (*R* = 0.300, *P* < .001), APRI (*R* = 0.174, *P* = .036), AAR (*R* = 0.222, *P* = .007), hepatic echo (*R* = 0.279, *P* = .001). The correlation between LS and the stage of liver fibrosis was the highest.

### 3.3. Results of multivariable analysis for prediction of liver cirrhosis

The significant indexes of univariate analysis were included in the binary logistic regression analysis after excluding collinearity (Table 2), and it was found that LS, splenomegaly, C4 and GP73 were the independent risk factors to predict AIH cirrhosis. Considering lost data missing, GP73 was not further used in model construction, other parameters will be included in the subsequent modeling analysis.

**Table 2:** Multivariable analysis result.

Index	β	*P*-value	OR	OR (95%CI)	
				Lower	Upper
ALB	−0.123	.21	0.884	0.729	1.072
PLT	0.000	.936	1.000	0.989	1.012
TBIL	0.007	.316	1.007	0.993	1.022
PT	0.714	.140	2.041	0.791	5.272
C4	−16.328	.009	0.020	0.010	0.040
ALP	−0.001	.493	0.999	0.994	1.003
BMI	−0.152	.376	0.859	0.614	1.203
Right-lobe-size	2.002	.140	7.401	0.519	105.460
Liver echo	0.185	.698	1.203	0.472	3.069
LS	0.348	.015	1.416	1.069	1.875
Splenomegaly	2.346	.006	10.446	1.959	55.697
Constant	−4.101	.640	0.017		

ALB = albumin; ALP = alkaline phosphatase; BMI = body mass index; C4 = complement C4; LS = liver stiffness; PLT = platelet; PT = prothrombin time; TBIL = total bilirubin.

### 3.4. Visual display of noninvasive prediction model

**Figure 3.**
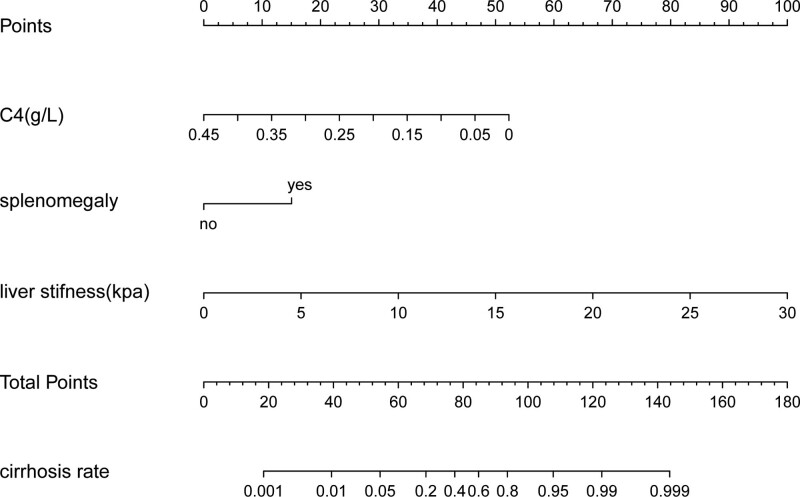
AIHC liver cirrhosis prediction model visualization. Example: when a patient has a C4 of 0.2, a score of about 40, splenomegaly, a score of about 15, and a liver hardness of 10, a score of about 30. The total score is 85, and the probability of cirrhosis is 60%. AIHC = autoimmune hepatitis cirrhosis.

Using R software, a Nomogram model was constructed, namely AIHC (Fig. 3). The risk score of all patients was calculated, and the Youden index was used to find the best cutoff value. In this study, 65 points was used as the best cutoff value, at this time, the diagnostic sensitivity was 88.9%, and the specificity was 75.6%.

### 3.5. Display of the calibration curve of the prediction model

**Figure 4.**
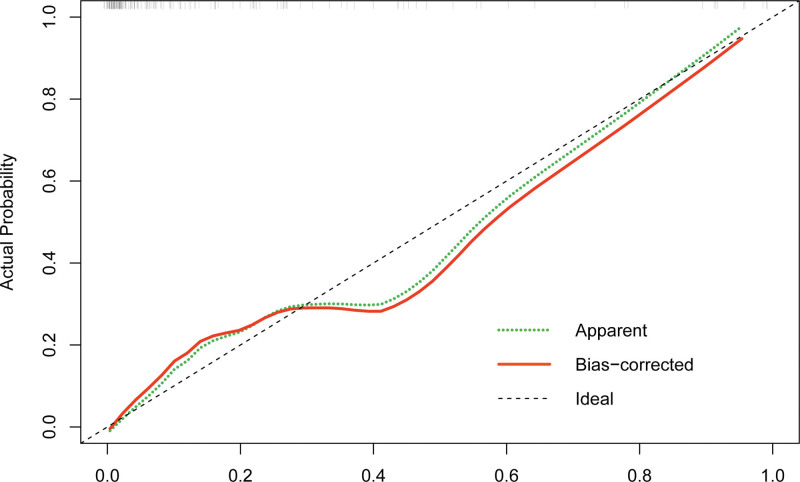
AIHC calibration curve. The prediction curve is distributed along the 45° line, indicating that the prediction accuracy is high. AIHC = autoimmune hepatitis cirrhosis.

The predicted probabilities are distributed along the 45° line (Fig. 4) with high accuracy.

### 3.6. ROC display of prediction model and other noninvasive indexes

**Figure 5.**
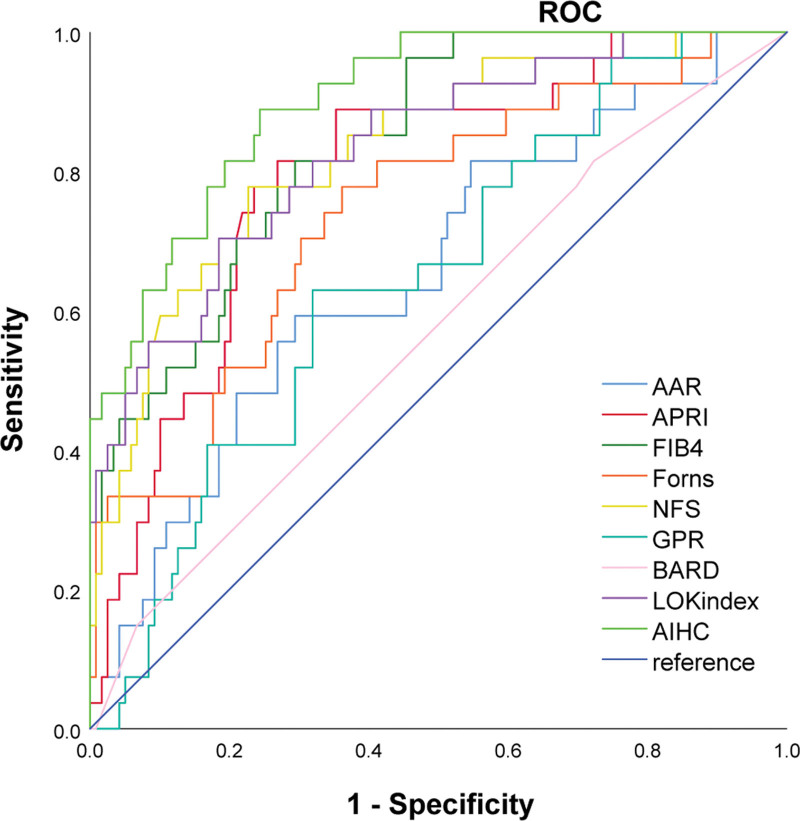
ROC curve of each index. AIHC has the largest area under the curve. AIHC = autoimmune hepatitis cirrhosis, ROC = receiver operating characteristic.

As shown in the ROC curve (Fig. 5) and the area under the curve (Table 3), the area under curve (AUC) of AIHC was significantly better than that of other indicators by Delong test, and the difference was statistically significant.

**Table 3:** AUC of each index.

Index	AUC	95% CI	
		Lower	Upper
AAR	0.656	0.541	0.771
APRI	0.795	0.706	0.884
FIB4	0.838	0.764	0.912
Forns	0.742	0.637	0.847
NFS	0.830	0.744	0.916
GPR	0.646	0.538	0.754
BARD	0.568	0.447	0.688
LOKindex	0.831	0.745	0.917
AIHC	0.901	0.845	0.957

### 3.7. Clinical decision curve presentation of predictive model and other noninvasive indicators

**Figure 6.**
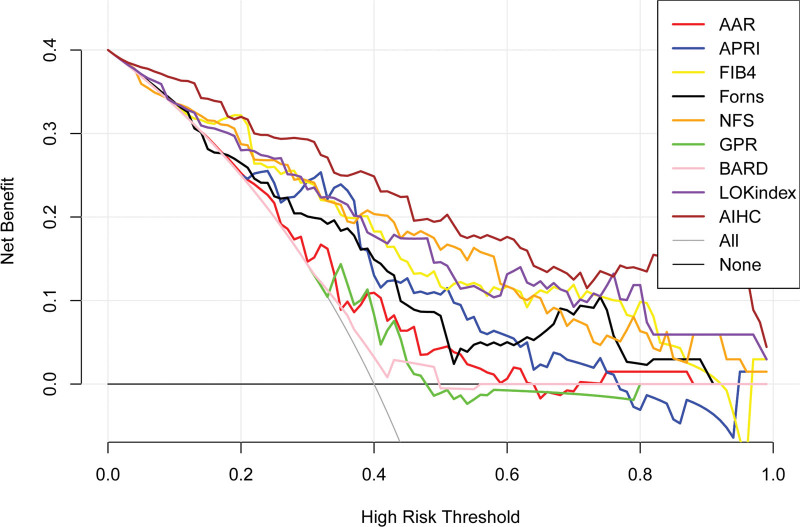
Clinical decision curve for each index. AIHC has the largest net benefit compared with other indicators. AIHC = autoimmune hepatitis cirrhosis.

The decision curve of each indicator (Fig. 6), from which we can see that AIHC has the largest benefit rate. The difference of NRI and integrated discrimination improvement (IDI) between AIHC and other indicators (Table 4) shows that AIHC has better NRI and IDI than the other indicators. If the NRI or IDI value was >1, that means the index was superior than AIHC otherwise inferior.

**Table 4:** NRI and IDI adjustment of AIHC compared with other indicators.

Index	NRI	Test value	*P*-value	IDI	Test value	*P*-value
AAR	0.347	2.664	.008	0.212	2.481	.013
APRI	0.099	0.854	.393	0.079	0.809	.419
FIB4	0.124	1.15	.25	0.1	1.918	.055
Forns	0.237	2.063	.039	0.17	3.333	.001
NFS	0.094	0.756	.45	0.064	0.626	.531
GPR	0.335	2.368	.018	0.21	2.297	.022
BARD	0.553	5.257	<.001	0.265	3.364	.001
Lokindex	0.126	1.035	.301	0.07	0.698	.485

IDI = integrated discrimination improvement, NRI = net reclassification index.

## 4. Discussion

Our research group collected 146 cases who met the requirements. Considering inflammation affect LS, only patients with inflammation level 0/1 were crewed in this study. Previous studies found that ultrasound LS measurement had a higher correlation with AIH fibrosis grade than other conventional indicators. Subsequently, the author fused ultrasound indicators with serological indicators to construct a noninvasive prediction model, AIHC, and visualized the model to obtain the best cutoff value for the diagnosis of cirrhosis. Compared with other commonly used noninvasive predictors, AIHC showed obvious advantages in AUC, NRI, IDI, and clinical decision curve.

Ultrasonography, as a routine screening method for liver lesions, is widely used in clinic to make a preliminary judgment of the degree of liver fibrosis by observing the echo of liver capsule and parenchyma, gallbladder wall, portal vein diameter, collateral vessels, spleen size, and so on. In normal liver tissue, the shape of hepatic sinusoids is regular, the fibrous matrix is arranged in order, and the echo is uniform. When the fibrous tissue around the hepatic sinusoids proliferates, or even pseudolobules are formed, the echo is uneven, with patchy, cord-like and even nodular echo. This is related to the disorder of parenchyma arrangement in the liver. Because of the disorder of the parenchyma in the liver, the adjacent capsule will also appear uneven, and in severe cases, the capsule show jagged changes. We did find the proportion of normal echo of liver parenchyma in the cirrhosis group was lower than that in the non-cirrhosis group, which was statistically significant. It was considered that the proportion of S2 and S3 in the non-cirrhosis group was higher. Although S2 and S3 patients did not meet the diagnostic criteria for cirrhosis, the structure of hepatic sinusoids had abnormal morphological changes. At this stage, the fibrous tissue around the hepatic sinusoid was also significantly increased compared with the normal liver tissue.

In this study, we did not find any difference in portal vein diameter between the 2 groups. The spleen is the largest parenchymal blood storage organ in the human body. When the splenic venous reflux is obstacle, it will lead to splenic parenchymal hyperplasia. The longer the time of liver parenchyma lesions, the more serious the splenic hyperplasia. In some patients with long-term illness, the diameter of the spleen can even exceed 20 cm. Therefore, the size of the spleen can often reflect the duration and severity of liver lesions. When the liver is normal, the lymphatic reflux of the gallbladder wall is unobstructed, and when the liver is cirrhosis, the lymphatic reflux is blocked, resulting in rough gallbladder wall and even edema of the gallbladder wall. A study by Montazeri showed that nodular changes in the liver capsule, widening of the hepatic fissure, thickening of the echo of the liver parenchyma, and splenomegaly may indicate the possibility of AIH cirrhosis.^[10]^ In another Han study, increased spleen size, rough and nodular liver capsule, and uneven echogenicity of liver parenchyma were also suggested as risk factors for cirrhosis.^[11]^ These findings are largely similar to those of the present study. In the multivariable analysis, only spleen size was ultimately considered as an independent risk factor for the development of cirrhosis, probably due to the reasons described above.^[13]^

Ultrasound elastography, as a new technology in nowadays, can estimate the degree of liver cirrhosis in a noninvasive manner, and has made outstanding contributions in the fields of hepatitis B, hepatitis C, alcoholic liver disease and nonalcoholic fatty liver disease. It has also found the best cutoff value.^[9,14]^ The use of elastic techniques in AIH is also increasing, and in this study, we also found a positive correlation between LS measurements and liver fibrosis grades in previous studies, which is consistent with many other studies.^[12,15]^ Maciej used ultrasound elastography (transient elastography, TE and two-dimensional shear wave elasticity) to measure liver and spleen stiffness to predict the incidence of AIH cirrhosis. When the cutoff value of liver and spleen stiffness was set at 16.1 kPa and 29.8 kPa, respectively, the AUC could reach 0.93 to 0.95. The study set the stiffness value too high, and the specificity was significantly improved. However, the sensitivity is relatively low, and for some unknown reasons, the same elastic measurement technology is not used in this study, which inevitably leads to measurement errors.^[16]^ In contrast, the same elasticity measurement technique was used throughout the study, and the same instrument was used throughout the study. The personnel performing the measurement were limited to a few experienced sonographers, so the stability was stronger and there were less errors.

Complement system is an important component of innate immune system, and also an important immune response substance against infection. However, patients with AIH suffer from disorder of immune system, where their autoantibodies are produced, and the content of complement in serum is increased accordingly. As an important auxiliary index of AIH, complement C4 plays an important role in the diagnosis of AIH.^[17]^ However, in the hardening stage of AIH, the synthesis ability of hepatocytes decreased, resulting in a significant decrease in various proteins. In this study, C4 and ALB in the hardening group were significantly lower than those in the nonhardening group. In the multivariable analysis, only C4 could be used as an independent risk factor for AIH hardening. It was considered that C4 was often significantly increased in the early stage of AIH. C4 is significantly lower, resulting in a more significant difference. ALB was at a low level in all stages of AIH, and the difference was not as obvious as C4. In addition to the complement system, the cytosolic DNA sensing cGAS-STING pathway has also been implicated in the pathogenesis of various autoimmune diseases.^[18]^ This pathway detects cytosolic DNA from damaged cells, triggering inflammatory responses. Although the involvement of cGAS-STING signaling in AIH is still unclear, it represents an intriguing area for further research. Dysregulated responses by this pathway could contribute to the aberrant autoimmunity and inflammation characterizing AIH. Further studies elucidating the status of cGAS-STING activation in AIH models and patients could provide valuable insights into disease mechanisms and therapeutic targets.

GP73 is a Golgi-resident transmembrane protein, which plays an important role in maintaining the normal function of human body. GP73 is widely distributed in body tissues, but it is particularly closely related to liver diseases. In previous studies, we have found that GP73 is positively expressed in both acute and chronic hepatitis, and the expression level is positively correlated with the severity of the disease.^[19]^ In Iftikhar study, GP73 was found to be actively expressed in activated stellate cells and was positively correlated with the degree of liver fibrosis.^[20]^ This is similar to the results of this study. In another Gateslis study, we also found a significant correlation between GP73 and the degree of liver fibrosis.^[21]^ Because there are too many missing data for this indicator, we did not include it in the prediction model.

## 5. Limitations

Although we have constructed a simple visualization model in this study, which is worthy of further promotion in clinical practice, there are still a few shortcomings. Firstly, this study is a retrospective study, and there was inevitably a selection bias. Secondly, there is some missing data in this study, as the total number of cases is scarce, so the mean interpolation method is used to supplement the data. The supplementary data is mainly GP73, in the final regression results, GP73, OR value is 1.014, although it has little impact on the final model, it is still inadequate. Thirdly, among the population we included, there are relatively few cases of patients with liver cirrhosis, which may potentially impact the robustness of the model. Finally, the model has only been verified by internal resampling, and its external applicability is still unknown. Therefore, it is crucial to increase the sample size by including more patients with liver cirrhosis to enhance the robustness of the model. Additionally, collaborating with external centers for external validation will be particularly important.

## 6. Conclusion

In this study, ultrasound indicators and serological indicators were organically combined to not only construct a visual model, but also find the best diagnostic threshold for judging AIH cirrhosis. Compared with other noninvasive indicators, it brings higher predictive accuracy and is worthy of further promotion in clinical practice.

## Author contributions

**Conceptualization:** Siyi Feng.

**Data curation:** Haibin Tu.

**Investigation:** Haibin Tu, Lihong Chen.

**Methodology:** Lihong Chen.

**Project administration:** Siyi Feng, Lihong Chen.

**Resources:** Siyi Feng.

**Software:** Siyi Feng.

**Supervision:** Siyi Feng.
